# Impact of the stress hyperglycemia ratio on short-term outcomes in critically ill patients with chronic kidney disease: A comparative analysis of diabetic and non-diabetic populations

**DOI:** 10.1371/journal.pone.0344961

**Published:** 2026-04-08

**Authors:** Xia Li, Zhikun Zhao, Xinyuan Zhang, Wenhua Yan, Dacheng Wang, Xue He, Chunjian Xu, Long Zhao, Haichen Yang

**Affiliations:** 1 Department of Geriatrics, The Affiliated Huaian Hospital of Xuzhou Medical University, Huaian, Jiangsu, China; 2 Department of Emergency Medicine, The Affiliated Huaian Hospital of Xuzhou Medical University, Huaian, Jiangsu, China; 3 Department of Intensive Care Unit, Affiliated Hospital of Nanjing University of Chinese Medicine, Nanjing, Jiangsu, China; 4 Department of Neurosurgery, Chenzhou third people’s Hospital, Chenzhou, Hunan, China‌‌; 5 Department of Anesthesia Surgery, The Affiliated Huaian No.1 People’s Hospital of Nanjing Medical University, Huaian, Jiangsu, China; King Faisal University College of Veterinary Medicine and Animal Resources, SAUDI ARABIA

## Abstract

**Background:**

Stress-induced hyperglycemia is common in critically ill patients and may worsen outcomes, especially in those with chronic kidney disease (CKD). The stress hyperglycemia ratio (SHR), defined as admission glucose divided by the estimated average glucose from HbA1c, reflects acute relative hyperglycemia, but its prognostic value in CKD remains unclear.

**Objective:**

To investigate the impact of SHR on ICU and 28-day mortality in critically ill CKD patients and to compare prognostic differences between diabetic and non-diabetic populations.

**Methods:**

Data of 1,836 CKD patients from the MIMIC-IV database were analyzed. SHR was categorized into quartiles, and patients were stratified by diabetes status. Kaplan–Meier survival analysis assessed ICU and 28-day mortality; Cox proportional hazards and restricted cubic spline (RCS) analyzes evaluated associations between SHR and outcomes. Subgroup analyzes explored effect modification by age, sex, and comorbidities.

**Results:**

In non-diabetic patients, higher SHR levels were independently associated with increased ICU and 28-day mortality. The adjusted hazard ratios (HRs) for ICU and 28-day mortality in the highest SHR quartile were 3.19 (95% CI: 1.60–6.53, P < 0.001) and 1.93 (95% CI: 1.14–3.27, P = 0.015), respectively. In diabetic patients, similar upward trends were observed, but the associations were not statistically significant after multivariate adjustment.

**Conclusions:**

Elevated SHR levels were associated with adverse short-term outcomes, particularly increased ICU and 28-day mortality in non-diabetic patients with chronic kidney disease. These findings suggest that stress-induced hyperglycemia may exert a harmful effect on critically ill patients without preexisting diabetes, emphasizing the importance of individualized glycemic management in this population.

## Introduction

Chronic kidney disease (CKD) is widespread globally, with 1.2 million deaths attributed to CKD in 2017 alone. Between 1999 and 2017, the global mortality rate from CKD increased by 41.4% [1]. In patients with CKD, diabetes is a common comorbidity, occurring in 30% to 40% of cases. It is closely associated with the progression of CKD and overall patient prognosis [2]. Although the overall incidence of CKD has slightly decreased in recent years, the prevalence of diabetes and diabetes-related CKD is on the rise, as is the global mortality rate from CKD,this trend has led to worse outcomes, especially in critically ill patients with CKD [3].

In the ICU, patients with chronic kidney disease (CKD), particularly those with critical illnesses, often develop stress hyperglycemia, characterized by acute elevations in blood glucose levels under physiological or pathological stress, which may normalize after the acute phase subsides [4]. Stress hyperglycemia is closely linked to alterations in hormone secretion, immune responses, and neural regulation. Epidemiological studies have shown that stress hyperglycemia occurs in approximately 30–60% of critically ill patients, and elevated stress hyperglycemia ratios (SHR) are observed in 25–40% of patients with acute coronary syndrome, 35–45% with acute stroke, and 40–50% of ICU patients with sepsis [4–7]. These findings highlight that stress-induced glucose dysregulation is both common and clinically relevant across multiple critical-care populations.

However, using a single measurement of admission blood glucose (ABG) to assess glycemic stress can be misleading, as it may be influenced by chronic glucose levels rather than reflecting the true magnitude of acute metabolic disturbance. To overcome this limitation, the stress hyperglycemia ratio (SHR) was introduced as a standardized metric that quantifies acute hyperglycemia relative to chronic glycemia [7]. SHR is calculated as admission glucose divided by the estimated average glucose derived from HbA1c. Accumulating evidence indicates that elevated SHR is an independent predictor of adverse outcomes in critically ill patients, with studies reporting substantially increased short-term mortality in acute coronary syndrome and acute stroke, as well as worse outcomes in sepsis and acute heart failure [7–13].

Importantly, the physiological differences between diabetic and non-diabetic CKD patients become particularly significant in critical care settings [14]. Diabetic patients, due to long-term metabolic disorders and reduced insulin secretion or sensitivity, are more susceptible to high blood sugar during stress; whereas non-diabetic patients, despite not having chronic metabolic issues, may also experience acute hyperglycemia during critical illness, leading to notably different prognoses [15,16]. Previous research has demonstrated significant differences in the correlation between the Stress Hyperglycemia Ratio (SHR) and adverse outcomes among diabetic and non-diabetic patients in the ICU. This establishes a foundation for further investigation into the prognostic value of SHR in patients with varying types of chronic kidney disease (CKD) [17].

This study aims to evaluate the impact of the Stress Hyperglycemia Ratio (SHR) on the prognosis of critically ill CKD patients, including both diabetic and non-diabetic individuals, in the ICU. This analysis aims to provide an evidence-based foundation for individualized glucose management strategies in critically ill patients with CKD.

## 1 Materials and methods

### 1.1 Study population and data source

The data for this study were obtained from the Medical Information Mart for Intensive Care IV (MIMIC-IV) database. To ensure patient privacy, all data were fully de-identified. The research team completed the National Institutes of Health (NIH) human research ethics training (Certification No. 55981932) and was granted authorized access to the database.

Patients with chronic kidney disease (CKD) were identified using the Charlson Comorbidity Index definitions in the MIMIC-IV database, which correspond to ICD-9 and ICD-10 diagnostic codes for CKD. Because consistent longitudinal eGFR data were not available, precise CKD staging could not be determined; therefore, all CKD patients were analyzed as a single group.

Only patients who were admitted to the ICU for the first time were included in the analysis. Patients without HbA1c data were excluded because HbA1c is required to calculate the stress hyperglycemia ratio (SHR). Other exclusion criteria included: multiple ICU admissions, age under 18 years, or ICU stays shorter than 24 hours..

### 1.2 Data collection and variable definitions

The baseline characteristics of all study patients were extracted from the MIMIC-IV database using PostgreSQL software, covering data from the first 24 hours after ICU admission.

These included demographic data (age and sex), disease severity scores (APS III and SOFA), and clinical interventions within 24 hours (such as continuous renal replacement therapy [CRRT]).

Comorbidities—including severe and mild liver disease, myocardial infarction, congestive heart failure, chronic pulmonary disease, and cerebrovascular disease—were identified using ICD-9 and ICD-10 diagnostic codes based on the Charlson Comorbidity Index definitions in the MIMIC-IV database. The complete ICD code definitions are publicly available in the official MIMIC-IV code repository on PhysioNet (https://github.com/MIT-LCP/mimic-code). Liver disease was defined as chronic hepatitis, cirrhosis, or hepatic failure.

Additional variables included arterial blood gas parameters (oxygen partial pressure, pH, oxygen saturation, and carbon dioxide partial pressure), vital signs (heart rate, mean arterial pressure, respiratory rate, temperature, and urine output), and a range of laboratory measurements (lactic acid, platelet count, white blood cell count, albumin, anion gap, blood urea nitrogen, creatinine, international normalized ratio, prothrombin time, partial thromboplastin time, alanine aminotransferase, aspartate aminotransferase, total bilirubin, creatine kinase-MB).

For SHR calculation, the admission blood glucose (ABG) was defined as the first available blood glucose measurement within 24 hours after ICU admission, representing the initial glycemic response to acute stress. The Stress Hyperglycemia Ratio (SHR) is calculated using the following formula: SHR = ABG (mg/dL)/ (28.7 × HbA1c (%)-46.7).

### 1.3 Outcome measures

The primary endpoints of this study are ICU mortality and 28-day overall mortality.

### 1.4 Ethics approval and consent to participate

The data used in this study came from public databases and cannot cause adverse effects on patients. Therefore, this study was exempted from ethical approval by the ethics committee of the Affiliated huaian Hospital of Xuzhou medical university.

### 1.5 Statistical analysis methods

Data analysis was performed using R (version 4.3.2) and Python (version 3.9) software. All statistical tests were two-sided, and a p-value < 0.05 was considered statistically significant. Continuous variables were first assessed for normality using the Kolmogorov–Smirnov test combined with histogram inspection. Variables that followed a normal distribution were expressed as mean ± standard deviation (SD) and compared using independent t-tests (Welch’s t-test when variances were unequal, and Student’s t-test when variances were equal). Non-normally distributed continuous variables were expressed as median (interquartile range, IQR) and compared using the Mann–Whitney U-test. Categorical variables were presented as frequencies and percentages and compared using the Pearson χ² test or Fisher’s exact test where appropriate, while ordered categorical variables were analyzed using the Mann–Whitney U-test. For multiple comparisons, p-values were adjusted using the Holm–Bonferroni method to control the family-wise error rate (FWER).

Initially, baseline differences between diabetic and non-diabetic groups were compared, followed by stratified analyzes based on the quartiles of SHR levels. Kaplan-Meier survival analysis with the log-rank test was used to evaluate differences in ICU and 28-day mortality rates between the groups. To further investigate the association between SHR levels and short-term outcomes, multivariable Cox proportional hazards models were constructed, adjusting for demographic characteristics, key comorbidities, and major clinical indicators. A sensitivity analysis was additionally performed after excluding the top 1% of SHR values to evaluate the influence of extreme outliers on the robustness of the results.

Restricted cubic spline (RCS) models with four knots located at the 5th, 35th, 65th, and 95th percentiles of SHR distribution were employed to explore potential non-linear relationships between SHR levels and ICU/28-day mortality. Non-linear associations were evaluated using the Wald test. After identifying potential inflection points from the RCS curves, piecewise linear regression models were constructed to estimate the hazard ratios (HRs) for SHR below and above the identified threshold.

Additionally, subgroup analyzes were conducted based on gender, age, APSIII scores, SOFA scores, CRRT treatment, and comorbidities such as myocardial infarction, congestive heart failure, and cerebrovascular disease.

## 2 Results

### 2.1 Comparison of baseline characteristics

This study included 1,836 ICU patients with chronic kidney disease, with a median age of 74 years (IQR 64–82), and 64.2% were male. Diabetic patients accounted for 57.7% of the cohort. Based on quartiles of the stress hyperglycemia ratio (SHR), patients were categorized into four groups: Q1 (0.37–1.15), Q2 (1.15–1.42), Q3 (1.42–1.73), and Q4 (1.73–15.04) (S1 Table).

Compared to non-diabetic patients, diabetic patients were younger, had higher APSIII scores, a greater use of CRRT, and a higher prevalence of myocardial infarction and congestive heart failure. They also exhibited significantly higher blood urea nitrogen and serum creatinine levels, and slightly lower INR values (all P < 0.05; S1 Table).

Across SHR quartiles, patient age, APSIII scores, and SOFA scores increased with rising SHR levels. Among non-diabetic patients, both ICU and 28-day mortality increased progressively with higher SHR levels. ICU mortality rose from 8.5% in Q1 to 19.2% in Q4, corresponding to an absolute difference of 10.7 percentage points (p = 0.007). Similarly, 28-day mortality increased from 11.5% to 20.4%, an absolute difference of 8.9 percentage points (p = 0.021). In contrast, among diabetic patients, although both ICU and 28-day mortality showed upward trends, the differences were not statistically significant (ICU mortality p = 0.082; 28-day mortality p = 0.240) (S2 Table)

### 2.2 Relationship between SHR and ICU/28-day mortality

**Fig 1:**
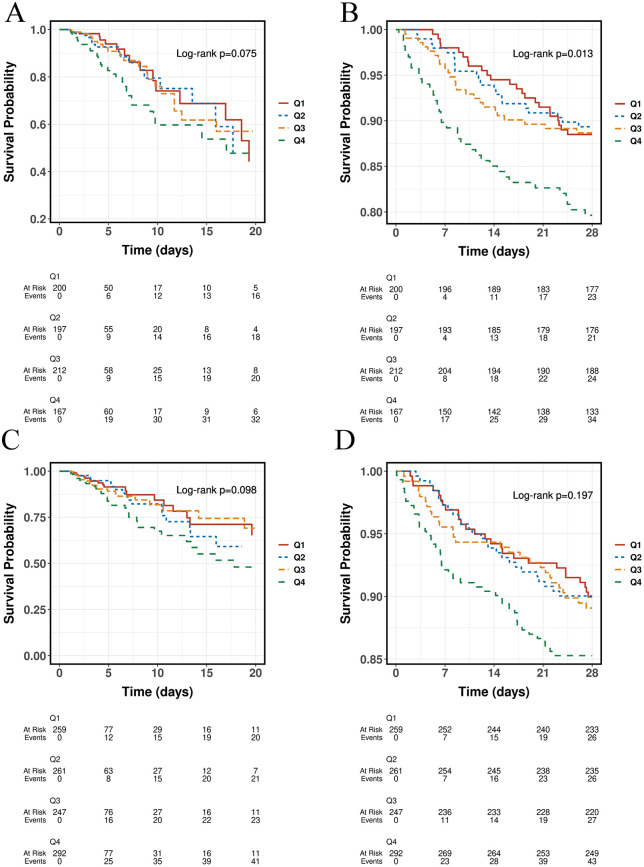
Kaplan-Meier survival curves for ICU mortality (A, C) and 28-day mortality (B, D) across different quartiles of the stress hyperglycemia ratio (SHR) in non-diabetic (A, B) and diabetic (C, D) patients. Survival differences were evaluated using the log-rank test.

**Fig 2:**
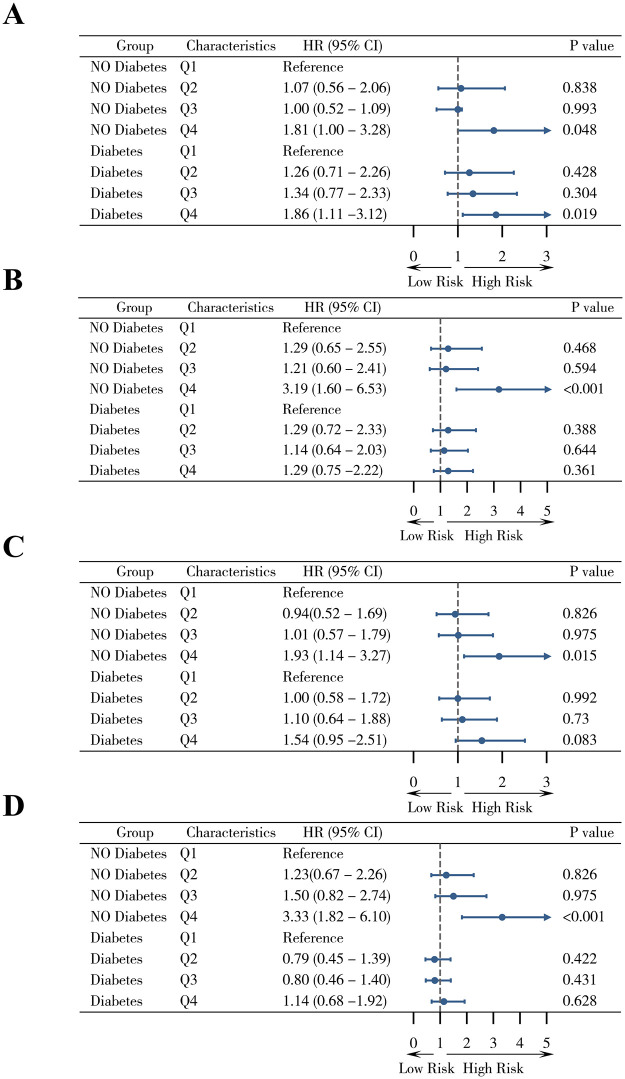
Forest plots showing the associations between SHR quartiles and ICU mortality (A, B) and 28-day mortality (C, D) in non-diabetic and diabetic patients. Hazard ratios (HRs) and 95% confidence intervals (CIs) were estimated using univariate (A, C) and multivariate (B, D) Cox proportional hazards models.

Kaplan-Meier survival analyzes were performed to evaluate ICU and 28-day mortality rates across different SHR levels in diabetic and non-diabetic patients (Figs 1A–2D). Among non-diabetic patients, ICU mortality showed a trend toward higher risk with increasing SHR levels, although the difference did not reach statistical significance (P = 0.075; Fig 1A). In contrast, 28-day mortality was significantly higher in patients with elevated SHR levels, particularly in the highest quartile (Q4) group (P = 0.013; Fig 1B). In diabetic patients, similar upward trends in ICU and 28-day mortality were observed with increasing SHR levels; however, these differences were not statistically significant (ICU mortality: P = 0.098, Fig 1C; 28-day mortality: P = 0.197, Fig 1D).

In non-diabetic patients, both ICU and 28-day mortality increased significantly with higher SHR levels. After multivariable adjustment, the highest SHR quartile (Q4) remained independently associated with ICU mortality (adjusted HR 3.19, 95% CI 1.60–6.53, P < 0.001) and 28-day mortality (adjusted HR 3.33, 95% CI 1.82–6.10, P < 0.001), indicating a robust relationship between stress hyperglycemia and short-term outcomes (Figs 2A–2D). In diabetic patients, similar upward trends were observed across SHR quartiles. However, after adjustment, the associations with ICU (adjusted HR 1.29, 95% CI 0.75–2.22, P = 0.361) and 28-day mortality (adjusted HR 1.14, 95% CI 0.68–1.92, P = 0.628) were not statistically significant (Figs 2A–2D).

After excluding the top 1% of SHR values, the results remained consistent with the main analysis. In non-diabetic patients, the highest SHR quartile (Q4, SHR 1.73–3.81) was still significantly associated with increased ICU (adjusted HR 3.03, 95% CI 1.51–6.09, P = 0.002) and 28-day mortality (adjusted HR 2.82, 95% CI 1.53–5.22, P < 0.001), whereas no significant associations were observed in diabetic patients (Supplementary S3 Table).

**Fig 3:**
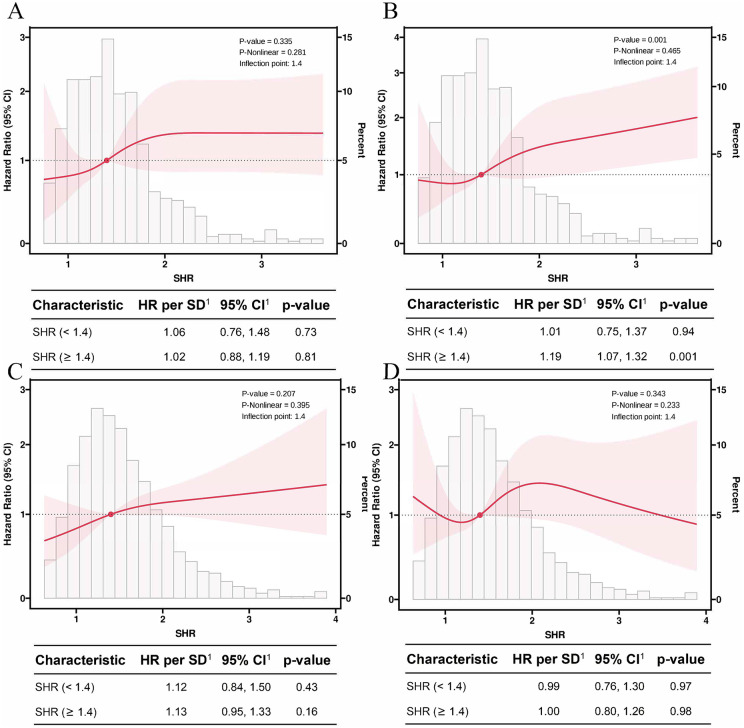
Restricted cubic spline (RCS) curves illustrating the relationship between SHR levels and ICU mortality (A, C) and 28-day mortality (B, D) in non-diabetic (A, B) and diabetic (C, D) patients. Solid lines represent the estimated hazard ratios, and shaded areas represent 95% confidence intervals.

The restricted cubic spline (RCS) analysis revealed that in non-diabetic patients (Figs 3A and 3B), both ICU and 28-day mortality rates exhibited an upward trend with increasing SHR levels. Specifically, when SHR exceeded 1.4, the 28-day mortality risk increased significantly (HR: 1.19, 95% CI: 1.07–1.32, P = 0.001). However, the overall association between SHR and ICU mortality did not reach statistical significance (P = 0.335), and no significant non-linear relationship was observed (P-nonlinear = 0.281). Similarly, for 28-day mortality, although an increasing trend was observed, the non-linearity was not statistically significant (P-nonlinear = 0.465). In diabetic patients (Figs 3C and 3D), increasing SHR levels also showed a trend towards higher ICU and 28-day mortality rates. However, neither the overall associations (ICU mortality: P = 0.207; 28-day mortality: P = 0.343) nor the non-linear trends (P-nonlinear = 0.395 and 0.233, respectively) reached statistical significance. These findings suggest that the prognostic impact of SHR appears to be more pronounced in non-diabetic patients compared to diabetic patients.

### 2.3 Subgroup analyzes

**Fig 4:**
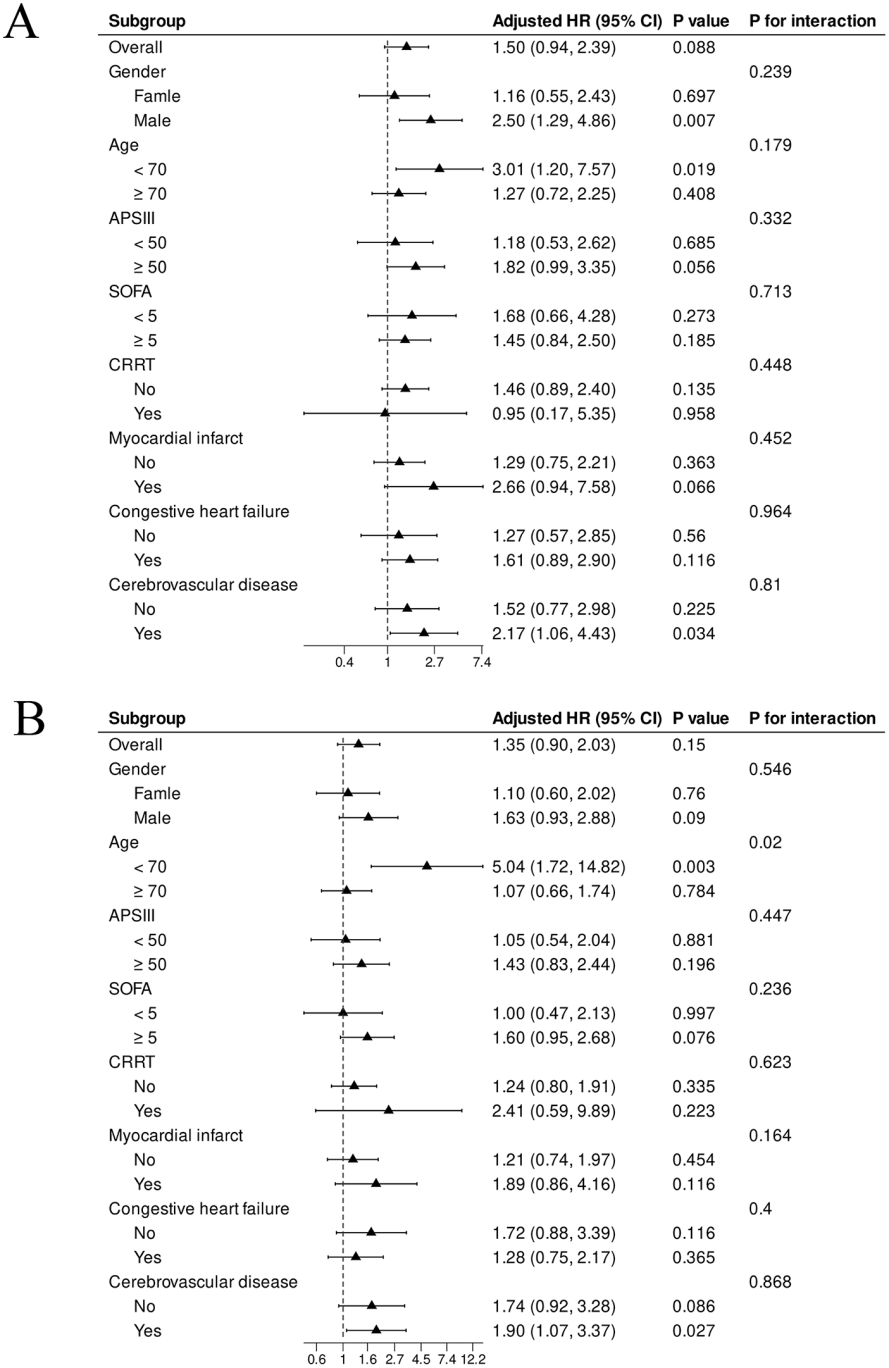
Subgroup analyzes of the association between SHR and ICU mortality (A) and 28-day mortality (B) in non-diabetic patients. Forest plots display hazard ratios (HRs) and 95% confidence intervals (CIs) across subgroups, with interaction P-values indicating the significance of effect modification.

For ICU mortality, although a trend toward higher risk with increasing SHR levels was observed across several subgroups—particularly among male patients, patients aged <70 years, and those with cerebrovascular disease—no statistically significant interactions were identified (all P for interaction > 0.05; Fig 4A). In the analysis of 28-day mortality, a significant interaction was found with age: the association between SHR and mortality was significantly stronger among patients aged <70 years compared to those aged ≥70 years (P for interaction = 0.020; Fig 4B). Additionally, while SHR was significantly associated with increased 28-day mortality risk among patients with cerebrovascular disease (HR: 1.90, 95% CI: 1.07–3.37, P = 0.027), no statistically significant interaction was observed for cerebrovascular disease status itself (P for interaction = 0.868). In diabetic patients, no significant interactions were detected for either ICU or 28-day mortality across the examined subgroups (S1 Fig).

## 3 Discussion

This study evaluated the impact of the Stress Hyperglycemia Ratio (SHR) on ICU and 28-day mortality in patients with chronic kidney disease (CKD) and further compared the differences between diabetic and non-diabetic patients. We found that in non-diabetic patients, higher SHR levels were independently associated with an increased risk of both ICU and 28-day mortality, and the restricted cubic spline (RCS) analysis further demonstrated that when SHR exceeded 1.4, the risk of 28-day mortality rose significantly. In contrast, among diabetic patients, although similar upward trends were observed, the associations between SHR and mortality did not reach statistical significance after adjustment.

Stress Hyperglycemia Ratio (SHR) has been widely recognized as a robust indicator of stress-related hyperglycemia, reflecting the imbalance between acute and chronic glycemic states. Previous studies have linked elevated SHR with adverse cardiovascular and metabolic outcomes in critically ill populations [8–10]. More recently, studies focusing on chronic kidney disease (CKD) populations have extended this evidence, showing that higher SHR levels are independently associated with increased short-term mortality and poor clinical outcomes [18]. Our findings further corroborate these observations, demonstrating that in CKD patients, the risk of both ICU and 28-day mortality increased significantly with higher SHR, particularly among non-diabetic individuals. This differential association between diabetic and non-diabetic patients is consistent with prior reports suggesting that long-term glycemic adaptation in diabetes may blunt the adverse effects of acute hyperglycemia [19].

The first possible explanation is that diabetes itself may lead to poor long-term outcomes, overshadowing the impact of high SHR [20]. Diabetic patients, having been exposed to chronically elevated glucose levels, may have adapted to these metabolic disturbances, making them less sensitive to acute glucose fluctuations [4]. Chronic hyperglycemia suppresses acute counter-regulatory responses by altering hypothalamic–pituitary–adrenal (HPA) axis sensitivity and impairing sympathetic activation, leading to blunted hormonal and metabolic reactions during acute stress [21]. The second possibility is that diabetic patients are in a constant state of inflammation and oxidative stress, while non-diabetic patients with newly developed hyperglycemia may exhibit a more intense inflammatory response than known diabetics [4]. In acute stress, rapid surges in catecholamines, cortisol, and pro-inflammatory cytokines (such as IL-6 and TNF-α) activate hepatic gluconeogenesis and induce transient but severe insulin resistance via the IRS-1/PI3K/AKT signaling pathway, contributing to endothelial dysfunction and mitochondrial injury [22]. In contrast, chronic low-grade inflammation in diabetes might lead to receptor desensitization and less pronounced cytokine responses.

Additionally, some evidence suggests that chronic hyperglycemia (as opposed to intermittent hyperglycemia) may establish a protective cellular regulatory mechanism, such as preferential downregulation of glucose transporters (GLUT-1 and GLUT-4) to reduce glucotoxicity, as well as enhancement of antioxidative enzyme systems (superoxide dismutase, catalase), making diabetic patients more resistant to acute glucose surges [23,24].

The third possibility is the metabolic memory effect, where poor long-term glycemic control in diabetic patients may induce persistent epigenetic changes, including DNA methylation and histone modification of genes involved in inflammation and fibrosis [25]. Even when glucose levels are controlled in the short term, these changes continue to affect gene expression through sustained activation of NF-κB and TGF-β pathways, leading to persistent secretion of inflammatory and fibrotic mediators [14]. These “metabolic memory” effects could explain why, even with elevated SHR, the increased prognostic risk in diabetic patients is less pronounced [26].

Although our study showed that 28-day mortality in diabetic CKD patients did not reach statistical significance, we observed a trend of increased mortality risk as SHR levels rose. This trend suggests that high SHR may still adversely affect short-term outcomes in diabetic CKD patients [17]. Evidence from CKD cohorts likewise links elevated SHR with worse short-term outcomes, although the magnitude of association may vary by diabetes status and clinical context [18,27]. Therefore, considering that pre-existing microvascular or macrovascular disease in diabetic patients may affect outcomes related to strict glucose control, for CKD patients, stricter glycemic control remains key to achieving better clinical outcomes, regardless of diabetes status [4].

## 4 Limitations

This study has several limitations. First, it was a retrospective analysis based on a single-center database, which may introduce selection bias. Second, patients without HbA1c data were excluded because HbA1c is required to calculate the stress hyperglycemia ratio (SHR). However, this exclusion may introduce potential selection bias, as HbA1c is not routinely measured in all critically ill patients. Third, CKD identification was based on diagnostic codes using the Charlson Comorbidity Index definitions in the MIMIC-IV database, which may not perfectly capture disease severity. Because consistent longitudinal eGFR data were unavailable, precise CKD staging could not be performed; therefore, all CKD patients were analyzed as a single group. Future studies incorporating detailed renal function data are warranted to determine whether the prognostic value of SHR differs across CKD stages. Fourth, certain variables such as insulin use and oral glucose-lowering medications were not available, which may have limited our ability to fully adjust for glycemic control. Fifth, the distribution of SHR in critically ill patients was right-skewed, resulting in a relatively wide upper range for the highest quartile (Q4: 1.73–15.04). Although this reflects the physiologic variability of stress responses, the wide range could potentially introduce minor instability in the upper category. To address this concern, a sensitivity analysis excluding the top 1% of SHR values was performed, and the results remained consistent with the main findings, confirming the robustness of the conclusions.

Finally, the study population was limited to ICU patients from a single database, and external validation using multi-center cohorts is needed to confirm the generalizability of these findings.

## 5 Conclusion

In critically ill patients with chronic kidney disease, higher SHR levels were significantly associated with increased ICU and 28-day mortality, particularly among non-diabetic individuals. In diabetic patients, similar upward trends in mortality risk were observed across SHR levels, although these associations did not reach statistical significance after adjustment. Restricted cubic spline analysis further demonstrated that the risk of 28-day mortality increased markedly when SHR exceeded approximately 1.4.

These findings identify SHR as a simple and practical marker for short-term risk stratification in CKD patients and underscore the importance of individualized glycemic management tailored to diabetic status.

## Supporting information

S1 Table Baseline characteristics of ICU patients with chronic kidney disease categorized by diabetes status. (DOCX)

S2 Table Baseline characteristics of ICU patients with chronic kidney disease categorized by diabetes status and SHR quartiles. (DOCX)

S3 Table Cox regression after excluding top 1% of SHR values (sensitivity analysis). (DOCX)

S1 Fig Subgroup analyses of the association between the stress hyperglycemia ratio (SHR) and ICU and 28-day mortality in diabetic patients. A:Subgroup analyses of the association between the stress hyperglycemia ratio (SHR) and ICU mortality in diabetic patients. B:Subgroup analyses of the association between the stress hyperglycemia ratio (SHR) and 28-day mortality in diabetic patients. Forest plots show hazard ratios (HRs) and 95% confidence intervals (CIs) across different clinical subgroups. No significant interactions were observed.
(DOCX)
