# Supplementary material for: Impact of the stress hyperglycemia ratio on short-term outcomes in critically ill patients with chronic kidney disease: A comparative analysis of diabetic and non-diabetic populations
Source: PLoS One. 2026 Apr 8;21(4):e0344961. doi: 10.1371/journal.pone.0344961 (PMC13061211; doi:10.1371/journal.pone.0344961)
Supplement: S1 Table — (DOCX) [file pone.0344961.s001.docx]

**Supplementary Table S1:** Baseline characteristics of ICU patients with chronic kidney disease categorized by diabetes status.

| **Variable** | **Overall (n=1835)** | **Non-diabetic (n=776)** | **Diabetic (n=1059)** | **p-value** |
| --- | --- | --- | --- | --- |
| Gender, n (%)¹ |  |  |  | 0.069 |
| Male | 1179 (64.25) | 517 (66.62) | 662 (62.51) |  |
| Female | 656 (35.75) | 259 (33.38) | 397 (37.49) |  |
| Age, years, Mean±SD⁴ | 69.46±13.36 | 71.62±13.70 | 67.88±12.89 | <0.001 |
| APSIII, score, M (Q1, Q3)³ | 50.00 (40.00, 61.00) | 47.00 (39.00, 59.00) | 51.00 (42.00, 62.00) | <0.001 |
| SOFA, score, M (Q1, Q3)³ | 6.00 (4.00, 8.00) | 6.00 (4.00, 8.00) | 6.00 (4.00, 8.00) | 0.491 |
| CRRT within 24 h, n (%)¹ |  |  |  | 0.002 |
| No | 1624 (88.50) | 708 (91.24) | 916 (86.50) |  |
| Yes | 211 (11.50) | 68 (8.76) | 143 (13.50) |  |
| Myocardial infarction, n (%)¹ |  |  |  | <0.001 |
| No | 1108 (60.38) | 508 (65.46) | 600 (56.66) |  |
| Yes | 727 (39.62) | 268 (34.54) | 459 (43.34) |  |
| Congestive heart failure, n (%)¹ |  |  |  | 0.052 |
| No | 789 (43.00) | 354 (45.62) | 435 (41.08) |  |
| Yes | 1046 (57.00) | 422 (54.38) | 624 (58.92) |  |
| Cerebrovascular disease, n (%)¹ |  |  |  | <0.001 |
| No | 1320 (71.93) | 517 (66.62) | 803 (75.83) |  |
| Yes | 515 (28.07) | 259 (33.38) | 256 (24.17) |  |
| Chronic pulmonary disease, n (%)¹ |  |  |  | 0.487 |
| No | 1335 (72.75) | 558 (71.91) | 777 (73.37) |  |
| Yes | 500 (27.25) | 218 (28.09) | 282 (26.63) |  |
| Mild liver disease, n (%)¹ |  |  |  | 0.098 |
| No | 1674 (91.23) | 698 (89.95) | 976 (92.16) |  |
| Yes | 161 (8.77) | 78 (10.05) | 83 (7.84) |  |
| Severe liver disease, n (%)¹ |  |  |  | 0.003 |
| No | 1780 (97.00) | 742 (95.62) | 1038 (98.02) |  |
| Yes | 55 (3.00) | 34 (4.38) | 21 (1.98) |  |
| pH, Mean±SD⁴ | 7.32±0.08 | 7.33±0.08 | 7.32±0.08 | 0.022 |
| SO₂, %, Mean±SD⁴ | 87.17±12.97 | 88.47±11.63 | 86.21±13.80 | <0.001 |
| PO₂, mmHg, M (Q1, Q3)³ | 82.80 (53.00, 113.00) | 86.20 (61.60, 121.00) | 80.00 (48.00, 110.10) | <0.001 |
| PCO₂, mmHg, Mean±SD⁴ | 47.04±9.89 | 47.02±10.56 | 47.05±9.38 | 0.952 |
| HR, bpm, Mean±SD⁴ | 81.42±13.48 | 81.41±13.70 | 81.44±13.32 | 0.964 |
| MBP, mmHg, Mean±SD⁴ | 58.29±13.96 | 58.95±14.26 | 57.81±13.73 | 0.085 |
| RR, bpm, Mean±SD⁴ | 27.91±6.03 | 27.77±6.21 | 28.02±5.90 | 0.374 |
| Temperature, °C, Mean±SD⁴ | 37.25±0.61 | 37.21±0.59 | 37.29±0.62 | 0.003 |
| Urine output, mL, M (Q1, Q3)³ | 1365.00 (783.50, 2068.00) | 1395.00 (850.00, 2126.25) | 1319.20 (750.00, 2040.00) | 0.042 |
| Lactate, mmol/L, M (Q1, Q3)³ | 2.40 (1.76, 3.23) | 2.40 (1.80, 3.20) | 2.40 (1.70, 3.30) | 0.674 |
| Platelets, ×10⁹/L, M (Q1, Q3)³ | 158.00 (114.00, 221.50) | 148.00 (108.00, 209.00) | 166.00 (120.50, 232.00) | <0.001 |
| WBC, ×10⁹/L, M (Q1, Q3)³ | 12.90 (9.50, 17.40) | 12.70 (9.47, 17.30) | 12.96 (9.50, 17.50) | 0.498 |
| Albumin, g/dL, Mean±SD⁴ | 3.38±0.44 | 3.42±0.42 | 3.36±0.45 | 0.003 |
| Anion gap , mmol/L, Mean±SD⁴ | 17.04±4.80 | 16.27±4.21 | 17.60±5.12 | <0.001 |
| BUN, mg/dL, M (Q1, Q3)³ | 35.00 (24.00, 52.00) | 31.00 (22.00, 46.00) | 38.00 (27.00, 56.00) | <0.001 |
| Creatinine, mg/dL, M (Q1, Q3)³ | 1.80 (1.40, 3.10) | 1.70 (1.30, 2.50) | 2.00 (1.50, 3.60) | <0.001 |
| INR, M (Q1, Q3)³ | 1.40 (1.20, 1.60) | 1.40 (1.20, 1.70) | 1.30 (1.20, 1.60) | 0.040 |
| PT, s, M (Q1, Q3)³ | 14.90 (13.00, 17.80) | 15.30 (13.10, 18.20) | 14.70 (12.90, 17.47) | 0.036 |
| PTT, s, M (Q1, Q3)³ | 35.10 (29.50, 55.25) | 35.60 (30.10, 54.92) | 34.80 (29.10, 55.70) | 0.104 |
| ALT, IU/L, M (Q1, Q3)³ | 27.80 (18.00, 58.00) | 26.40 (17.00, 49.30) | 29.20 (19.00, 63.60) | 0.001 |
| AST, IU/L, M (Q1, Q3)³ | 45.00 (27.40, 95.30) | 43.10 (27.00, 91.85) | 45.80 (28.00, 96.00) | 0.191 |
| Total bilirubin, mg/dL, M (Q1, Q3)³ | 0.56 (0.40, 0.80) | 0.60 (0.44, 0.88) | 0.52 (0.38, 0.71) | <0.001 |
| CK-CPK, U/L, M (Q1, Q3)³ | 195.00 (107.20, 516.30) | 171.20 (105.30, 507.25) | 211.00 (110.30, 518.00) | 0.138 |
| CK-MB, U/L, M (Q1, Q3)³ | 8.00 (4.60, 23.90) | 8.00 (4.40, 22.85) | 8.20 (4.60, 24.20) | 0.624 |
| Serum creatinine, mg/dL, M (Q1, Q3)³ | 1.30 (1.00, 1.90) | 1.20 (1.00, 1.70) | 1.30 (1.00, 2.10) | <0.001 |
| SHR, M (Q1, Q3)³ | 1.42 (1.15, 1.73) | 1.41 (1.14, 1.68) | 1.43 (1.15, 1.78) | 0.104 |
| ICU mortality, n (%)¹ |  |  |  | 0.761 |
| No | 1627 (88.66) | 686 (88.40) | 941 (88.86) |  |
| Yes | 208 (11.34) | 90 (11.60) | 118 (11.14) |  |
| 28-day mortality, n (%)¹ |  |  |  | 0.294 |
| No | 1611 (87.79) | 674 (86.86) | 937 (88.48) |  |
| Yes | 224 (12.21) | 102 (13.14) | 122 (11.52) |  |

Notes: **¹ Pearson χ² test; ² Welch’s t-test; ³ Mann–Whitney U test; ⁴ Student’s t-test.**

Continuous variables with normal distributions are expressed as mean ± SD and compared using Student’s t-test; non-normally distributed variables are expressed as median (IQR) and compared using the Mann–Whitney U test. Categorical variables are presented as n (%) and compared using the Pearson χ² or Fisher’s exact test, as appropriate.

Abbreviations: SHR, stress hyperglycemia ratio; APSIII, Acute Physiology Score III; SOFA, Sequential Organ Failure Assessment; CRRT, continuous renal replacement therapy; PO₂, partial pressure of oxygen; pH, potential of hydrogen; SO₂, oxygen saturation; PCO₂, partial pressure of carbon dioxide; HR, heart rate; MBP, mean blood pressure; RR, respiratory rate; T, temperature; WBC, white blood cells; INR, international normalized ratio; PT, prothrombin time; PTT, partial thromboplastin time; ALT, alanine aminotransferase; AST, aspartate aminotransferase; BUN, blood urea nitrogen; CK-CPK, creatine kinase (total creatine phosphokinase); CK-MB, creatine kinase MB isoenzyme; SD, standard deviation; IQR, interquartile range; M (Q1, Q3), median (interquartile range); Q, quartile; ICU mortality, intensive care unit mortality.
