# Supplementary material for: Impact of the stress hyperglycemia ratio on short-term outcomes in critically ill patients with chronic kidney disease: A comparative analysis of diabetic and non-diabetic populations
Source: PLoS One. 2026 Apr 8;21(4):e0344961. doi: 10.1371/journal.pone.0344961 (PMC13061211; doi:10.1371/journal.pone.0344961)
Supplement: S2 Table — (DOCX) [file pone.0344961.s002.docx]

**Supplementary Table 2:** Baseline characteristics of ICU patients with chronic kidney disease categorized by diabetes status and SHR quartiles.

|  | **Non-diabetic (n=776)** | | | | | **Diabetic (n=1059)** | | | | |
| --- | --- | --- | --- | --- | --- | --- | --- | --- | --- | --- |
| **Variable** | **Q1** | **Q2** | **Q3** | **Q4** | **p-value** | **Q1** | **Q2** | **Q3** | **Q4** | **p-value** |
| Gender, n (%)¹ |  |  |  |  | 0.777 |  |  |  |  | 0.154 |
| Male | 135 (67.50) | 127 (64.47) | 146 (68.87) | 109 (65.27) |  | 175 (67.57) | 154 (59.00) | 158 (63.97) | 175 (59.93) |  |
| Female | 65 (32.50) | 70 (35.53) | 66 (31.13) | 58 (34.73) |  | 84 (32.43) | 107 (41.00) | 89 (36.03) | 117 (40.07) |  |
| Age, years, Mean±SD⁴ | 71.19±14.64 | 73.83±12.17 | 71.40±14.38 | 69.82±13.10 | 0.040 | 66.56±13.01 | 68.93±12.68 | 69.94±12.20 | 66.38±13.28 | 0.002 |
| APSIII, score, M (Q1, Q3)³ | 43.00 (36.00, 53.00) | 46.00 (38.00, 58.00) | 48.00 (40.75, 59.00) | 55.00 (41.00, 69.00) | <0.001 | 48.00 (39.00, 60.00) | 50.00 (41.00, 60.00) | 52.00 (42.50, 62.00) | 53.00 (45.00, 63.00) | <0.001 |
| SOFA, score, M (Q1, Q3)³ | 4.00 (3.00, 6.00) | 6.00 (4.00, 8.00) | 6.00 (4.00, 8.00) | 7.00 (5.00, 9.50) | <0.001 | 5.00 (4.00, 7.00) | 6.00 (4.00, 7.00) | 6.00 (4.00, 8.00) | 6.00 (4.00, 8.00) | 0.154 |
| CRRT within 24 h, n (%)¹ |  |  |  |  | 0.356 |  |  |  |  | 0.007 |
| No | 185 (92.50) | 183 (92.89) | 193 (91.04) | 147 (88.02) |  | 237 (91.51) | 228 (87.36) | 213 (86.23) | 238 (81.51) |  |
| Yes | 15 (7.50) | 14 (7.11) | 19 (8.96) | 20 (11.98) |  | 22 (8.49) | 33 (12.64) | 34 (13.77) | 54 (18.49) |  |
| Myocardial infarction, n (%)¹ |  |  |  |  | 0.227 |  |  |  |  | 0.997 |
| No | 142 (71.00) | 125 (63.45) | 131 (61.79) | 110 (65.87) |  | 147 (56.76) | 148 (56.70) | 141 (57.09) | 164 (56.16) |  |
| Yes | 58 (29.00) | 72 (36.55) | 81 (38.21) | 57 (34.13) |  | 112 (43.24) | 113 (43.30) | 106 (42.91) | 128 (43.84) |  |
| Congestive heart failure, n (%)¹ |  |  |  |  | 0.933 |  |  |  |  | 0.543 |
| No | 94 (47.00) | 91 (46.19) | 96 (45.28) | 73 (43.71) |  | 110 (42.47) | 113 (43.30) | 102 (41.30) | 110 (37.67) |  |
| Yes | 106 (53.00) | 106 (53.81) | 116 (54.72) | 94 (56.29) |  | 149 (57.53) | 148 (56.70) | 145 (58.70) | 182 (62.33) |  |
| Cerebrovascular disease, n (%)¹ |  |  |  |  | <0.001 |  |  |  |  | 0.772 |
| No | 108 (54.00) | 123 (62.44) | 149 (70.28) | 137 (82.04) |  | 199 (76.83) | 195 (74.71) | 183 (74.09) | 226 (77.40) |  |
| Yes | 92 (46.00) | 74 (37.56) | 63 (29.72) | 30 (17.96) |  | 60 (23.17) | 66 (25.29) | 64 (25.91) | 66 (22.60) |  |
| Chronic pulmonary disease, n (%)¹ |  |  |  |  | 0.167 |  |  |  |  | 0.193 |
| No | 155 (77.50) | 133 (67.51) | 151 (71.23) | 119 (71.26) |  | 197 (76.06) | 192 (73.56) | 187 (75.71) | 201 (68.84) |  |
| Yes | 45 (22.50) | 64 (32.49) | 61 (28.77) | 48 (28.74) |  | 62 (23.94) | 69 (26.44) | 60 (24.29) | 91 (31.16) |  |
| Mild liver disease, n (%)¹ |  |  |  |  | 0.003 |  |  |  |  | 0.105 |
| No | 187 (93.50) | 182 (92.39) | 191 (90.09) | 138 (82.63) |  | 234 (90.35) | 246 (94.25) | 233 (94.33) | 263 (90.07) |  |
| Yes | 13 (6.50) | 15 (7.61) | 21 (9.91) | 29 (17.37) |  | 25 (9.65) | 15 (5.75) | 14 (5.67) | 29 (9.93) |  |
| Severe liver disease, n (%)¹ |  |  |  |  | 0.061 |  |  |  |  | 0.271 |
| No | 196 (98.00) | 189 (95.94) | 203 (95.75) | 154 (92.22) |  | 252 (97.30) | 259 (99.23) | 243 (98.38) | 284 (97.26) |  |
| Yes | 4 (2.00) | 8 (4.06) | 9 (4.25) | 13 (7.78) |  | 7 (2.70) | 2 (0.77) | 4 (1.62) | 8 (2.74) |  |
| pH, Mean±SD⁴ | 7.36±0.06 | 7.33±0.07 | 7.32±0.08 | 7.29±0.10 | <0.001 | 7.33±0.07 | 7.32±0.07 | 7.32±0.07 | 7.30±0.10 | <0.001 |
| SO₂, %, Mean±SD⁴ | 86.12±11.40 | 88.72±11.74 | 90.44±11.32 | 88.48±11.77 | 0.002 | 87.99±12.18 | 88.78±11.27 | 87.06±13.76 | 81.62±16.01 | <0.001 |
| PO₂, mmHg, M (Q1, Q3)³ | 90.80 (60.35, 131.10) | 86.40 (64.00, 114.00) | 88.00 (68.00, 121.25) | 80.00 (53.00, 105.50) | 0.091 | 83.20 (58.70, 113.00) | 86.00 (64.00, 122.00) | 82.00 (54.90, 111.10) | 63.00 (39.00, 95.00) | <0.001 |
| PCO₂, mmHg, Mean±SD⁴ | 45.70±10.20 | 47.19±10.62 | 47.46±11.32 | 47.86±9.82 | 0.202 | 46.70±7.96 | 46.46±8.75 | 47.18±9.38 | 47.79±10.95 | 0.354 |
| HR, bpm, Mean±SD⁴ | 80.41±14.93 | 80.80±12.99 | 81.71±13.42 | 82.93±13.32 | 0.309 | 80.43±13.69 | 80.91±12.46 | 82.42±12.78 | 81.96±14.14 | 0.301 |
| MBP, mmHg, Mean±SD⁴ | 62.80±15.18 | 58.10±14.25 | 58.12±14.48 | 56.40±11.87 | <0.001 | 59.47±13.73 | 58.51±14.69 | 56.41±12.89 | 56.90±13.40 | 0.040 |
| RR, bpm, Mean±SD⁴ | 28.54±6.61 | 27.77±6.15 | 27.84±6.60 | 26.74±5.08 | 0.052 | 27.57±5.62 | 27.91±5.86 | 28.03±5.99 | 28.51±6.09 | 0.301 |
| Temperature, °C, Mean±SD⁴ | 37.20±0.51 | 37.24±0.50 | 37.23±0.66 | 37.15±0.67 | 0.405 | 37.25±0.61 | 37.31±0.57 | 37.28±0.63 | 37.31±0.66 | 0.578 |
| Urine output, mL, M (Q1, Q3)³ | 1512.50 (825.00, 2266.25) | 1450.00 (945.00, 2190.00) | 1415.00 (1012.50, 2008.75) | 1165.00 (567.50, 1947.50) | 0.001 | 1320.00 (879.00, 2090.00) | 1290.00 (780.00, 1855.00) | 1275.00 (690.50, 1882.50) | 1390.00 (623.25, 2177.50) | 0.365 |
| Lactate, mmol/L, M (Q1, Q3)³ | 2.10 (1.59, 2.68) | 2.40 (1.80, 3.00) | 2.41 (1.90, 3.40) | 2.90 (2.10, 4.55) | <0.001 | 2.10 (1.59, 2.80) | 2.38 (1.80, 3.30) | 2.50 (1.70, 3.40) | 2.60 (1.72, 3.61) | <0.001 |
| Platelets, ×10⁹/L, M (Q1, Q3)³ | 190.50 (138.75, 247.50) | 148.00 (108.00, 211.00) | 141.50 (104.75, 184.75) | 125.00 (86.50, 174.00) | <0.001 | 161.00 (121.00, 232.00) | 167.00 (121.00, 219.00) | 160.00 (114.00, 228.50) | 175.00 (121.75, 239.50) | 0.567 |
| WBC, ×10⁹/L, M (Q1, Q3)³ | 10.35 (8.10, 13.43) | 12.50 (9.70, 16.70) | 13.85 (10.70, 18.30) | 15.60 (10.35, 20.10) | <0.001 | 12.40 (9.10, 16.35) | 12.70 (9.60, 17.40) | 13.10 (9.60, 18.45) | 13.45 (9.50, 17.60) | 0.288 |
| Albumin, g/dL, Mean±SD⁴ | 3.52±0.41 | 3.43±0.42 | 3.42±0.32 | 3.27±0.50 | <0.001 | 3.40±0.46 | 3.41±0.40 | 3.36±0.48 | 3.26±0.45 | <0.001 |
| Anion gap , mmol/L, Mean±SD⁴ | 16.48±3.54 | 15.38±3.83 | 16.11±4.06 | 17.29±5.23 | <0.001 | 16.65±4.52 | 16.86±4.58 | 17.28±4.85 | 19.36±5.83 | <0.001 |
| BUN, mg/dL, M (Q1, Q3)³ | 32.00 (23.00, 51.00) | 28.00 (21.00, 41.00) | 31.00 (21.00, 43.00) | 33.00 (22.50, 50.50) | 0.080 | 34.00 (24.50, 48.50) | 34.00 (25.00, 51.00) | 37.00 (26.00, 56.00) | 46.50 (33.00, 64.00) | <0.001 |
| Creatinine, mg/dL, M (Q1, Q3)³ | 1.70 (1.30, 2.50) | 1.60 (1.30, 2.20) | 1.60 (1.30, 2.30) | 2.00 (1.35, 3.20) | 0.023 | 1.90 (1.40, 3.10) | 1.80 (1.30, 3.00) | 2.00 (1.50, 3.60) | 2.50 (1.60, 4.45) | <0.001 |
| INR, M (Q1, Q3)³ | 1.30 (1.10, 1.50) | 1.40 (1.20, 1.60) | 1.40 (1.20, 1.70) | 1.50 (1.30, 1.80) | <0.001 | 1.30 (1.20, 1.60) | 1.40 (1.20, 1.62) | 1.30 (1.20, 1.60) | 1.30 (1.12, 1.60) | 0.469 |
| PT, s, M (Q1, Q3)³ | 13.80 (12.30, 16.33) | 15.10 (13.00, 17.70) | 15.40 (13.30, 18.20) | 16.20 (14.35, 19.40) | <0.001 | 14.70 (12.90, 17.15) | 15.00 (13.10, 17.80) | 14.70 (13.10, 17.18) | 14.35 (12.57, 17.90) | 0.473 |
| PTT, s, M (Q1, Q3)³ | 33.20 (28.70, 46.42) | 36.00 (30.50, 53.90) | 37.40 (30.87, 60.88) | 36.60 (30.65, 58.15) | 0.020 | 34.70 (29.95, 52.36) | 35.00 (30.00, 54.10) | 35.70 (28.60, 61.75) | 33.90 (28.48, 51.25) | 0.571 |
| ALT, IU/L, M (Q1, Q3)³ | 23.00 (15.00, 42.00) | 23.40 (15.60, 37.20) | 27.80 (18.00, 58.55) | 28.60 (20.00, 56.90) | 0.001 | 28.80 (17.00, 68.50) | 27.80 (19.00, 61.80) | 31.00 (20.40, 65.80) | 31.20 (18.00, 59.40) | 0.711 |
| AST, IU/L, M (Q1, Q3)³ | 34.20 (22.00, 77.85) | 40.20 (27.00, 74.60) | 49.80 (29.35, 118.00) | 52.00 (34.50, 100.40) | <0.001 | 43.00 (25.00, 100.00) | 45.80 (26.00, 97.20) | 48.00 (29.80, 100.30) | 45.80 (30.00, 82.25) | 0.597 |
| Total bilirubin, mg/dL, M (Q1, Q3)³ | 0.60 (0.42, 0.80) | 0.60 (0.48, 0.90) | 0.60 (0.49, 0.90) | 0.56 (0.40, 0.97) | 0.547 | 0.54 (0.40, 0.72) | 0.56 (0.40, 0.76) | 0.52 (0.40, 0.72) | 0.48 (0.32, 0.70) | 0.005 |
| CK-CPK, U/L, M (Q1, Q3)³ | 159.20 (106.15, 472.45) | 156.00 (102.00, 362.40) | 178.30 (102.75, 544.90) | 204.00 (118.60, 540.60) | 0.202 | 199.20 (100.50, 479.70) | 205.80 (104.00, 517.60) | 237.20 (124.00, 557.90) | 201.90 (110.00, 431.65) | 0.300 |
| CK-MB, U/L, M (Q1, Q3)³ | 5.10 (3.00, 12.20) | 7.80 (4.80, 19.40) | 9.70 (5.00, 41.40) | 9.60 (6.00, 51.80) | <0.001 | 8.00 (4.10, 23.30) | 8.00 (4.60, 20.20) | 9.00 (4.80, 37.00) | 8.00 (5.00, 22.05) | 0.253 |
| Serum creatinine, mg/dL, M (Q1, Q3)³ | 1.20 (1.00, 1.70) | 1.20 (1.00, 1.60) | 1.20 (0.90, 1.60) | 1.20 (1.00, 1.70) | 0.778 | 1.30 (1.00, 1.80) | 1.20 (1.00, 1.90) | 1.40 (1.10, 2.10) | 1.50 (1.10, 2.40) | <0.001 |
| ICU mortality, n (%)¹ |  |  |  |  | 0.007 |  |  |  |  | 0.082 |
| No | 183 (91.50) | 178 (90.36) | 190 (89.62) | 135 (80.84) |  | 237 (91.51) | 237 (90.80) | 218 (88.26) | 249 (85.27) |  |
| Yes | 17 (8.50) | 19 (9.64) | 22 (10.38) | 32 (19.16) |  | 22 (8.49) | 24 (9.20) | 29 (11.74) | 43 (14.73) |  |
| 28-day mortality, n (%)¹ |  |  |  |  | 0.021 |  |  |  |  | 0.240 |
| No | 177 (88.50) | 176 (89.34) | 188 (88.68) | 133 (79.64) |  | 233 (89.96) | 235 (90.04) | 220 (89.07) | 249 (85.27) |  |
| Yes | 23 (11.50) | 21 (10.66) | 24 (11.32) | 34 (20.36) |  | 26 (10.04) | 26 (9.96) | 27 (10.93) | 43 (14.73) |  |

Notes: **SHR levels: Q1 (0.37–1.15), Q2 (1.15–1.42), Q3 (1.42–1.73), Q4 (1.73–15.04).**

**¹ Pearson χ² test; ² Welch’s t-test; ³ Mann–Whitney U test; ⁴ Student’s t-test.**

Continuous variables with normal distributions are expressed as mean ± SD and compared using Student’s t-test; non-normally distributed variables are expressed as median (IQR) and compared using the Mann–Whitney U test. Categorical variables are presented as n (%) and compared using the Pearson χ² or Fisher’s exact test, as appropriate.

Abbreviations: SHR, stress hyperglycemia ratio; APSIII, Acute Physiology Score III; SOFA, Sequential Organ Failure Assessment; CRRT, continuous renal replacement therapy; PO₂, partial pressure of oxygen; pH, potential of hydrogen; SO₂, oxygen saturation; PCO₂, partial pressure of carbon dioxide; HR, heart rate; MBP, mean blood pressure; RR, respiratory rate; T, temperature; WBC, white blood cells; INR, international normalized ratio; PT, prothrombin time; PTT, partial thromboplastin time; ALT, alanine aminotransferase; AST, aspartate aminotransferase; BUN, blood urea nitrogen; CK-CPK, creatine kinase (total creatine phosphokinase); CK-MB, creatine kinase MB isoenzyme; SD, standard deviation; IQR, interquartile range; M (Q1, Q3), median (interquartile range); Q, quartile; ICU mortality, intensive care unit mortality.
