# Supplementary material for: Impact of the stress hyperglycemia ratio on short-term outcomes in critically ill patients with chronic kidney disease: A comparative analysis of diabetic and non-diabetic populations
Source: PLoS One. 2026 Apr 8;21(4):e0344961. doi: 10.1371/journal.pone.0344961 (PMC13061211; doi:10.1371/journal.pone.0344961)
Supplement: S3 Table — (DOCX) [file pone.0344961.s003.docx]

**Supplementary Table 3：** **Cox regression after excluding top 1% of SHR values (sensitivity analysis)**

| **Population** | **Outcome** | **SHR Quartile (after excluding top 1%)** | **HR (Unadjusted) (95% CI)** | **P value** | **HR (Adjusted) (95% CI)** | **P value** |
| --- | --- | --- | --- | --- | --- | --- |
| **Non-diabetic** | **ICU mortality** | Q1 (0.371–1.148) | Reference | — | Reference | — |
|  |  | Q2 (1.150–1.420) | 1.07 (0.55–2.06) | 0.846 | 1.28 (0.65–2.55) | 0.474 |
|  |  | Q3 (1.420–1.730) | 1.00 (0.52–1.90) | 0.995 | 1.18 (0.59–2.37) | 0.633 |
|  |  | **Q4 (1.73–3.81)** | 1.78 (0.98–3.25) | 0.059 | **3.03 (1.51–6.09)** | **0.002** |
|  | **28-day mortality** | Q1 (0.371–1.148) | Reference | — | Reference | — |
|  |  | Q2 (1.150–1.420) | 0.94 (0.52–1.69) | 0.825 | 1.20 (0.65–2.18) | 0.561 |
|  |  | Q3 (1.420–1.730) | 1.01 (0.57–1.79) | 0.975 | 1.22 (0.67–2.22) | 0.525 |
|  |  | **Q4 (1.73–3.81)** | **1.83 (1.07–3.14)** | **0.028** | **2.82 (1.53–5.22)** | **<0.001** |
| **Diabetic** | **ICU mortality** | Q1 (≈0.37–1.15) | Reference | — | Reference | — |
|  |  | Q2 (≈1.15–1.42) | 1.26 (0.71–2.26) | 0.430 | 1.30 (0.72–2.34) | 0.380 |
|  |  | Q3 (≈1.42–1.73) | 1.34 (0.77–2.33) | 0.304 | 1.15 (0.65–2.03) | 0.642 |
|  |  | **Q4 (1.73–3.81)** | **1.87 (1.11–3.14)** | **0.018** | 1.27 (0.74–2.19) | 0.392 |
|  | **28-day mortality** | Q1 (≈0.37–1.15) | Reference | — | Reference | — |
|  |  | Q2 (≈1.15–1.42) | 1.00 (0.58–1.72) | 0.992 | 0.79 (0.45–1.39) | 0.409 |
|  |  | Q3 (≈1.42–1.73) | 1.10 (0.64–1.88) | 0.730 | 0.80 (0.46–1.39) | 0.427 |
|  |  | **Q4 (1.73–3.81)** | 1.57 (0.96–2.55) | 0.073 | 1.14 (0.67–1.92) | 0.631 |

**Note:** Sensitivity analysis excluding patients with SHR >99th percentile (top 1%). Quartile categorization followed the original cutoffs; after exclusion, the **effective upper bound of Q4 reduced to 3.81,** and all Cox models were re-estimated on this restricted cohort. Adjusted models control for prespecified covariates as in the primary analysis.
