# Supplementary material for: Impact of the stress hyperglycemia ratio on short-term outcomes in critically ill patients with chronic kidney disease: A comparative analysis of diabetic and non-diabetic populations
Source: PLoS One. 2026 Apr 8;21(4):e0344961. doi: 10.1371/journal.pone.0344961 (PMC13061211; doi:10.1371/journal.pone.0344961)
Supplement: S1 Fig — A:Subgroup analyses of the association between the stress hyperglycemia ratio (SHR) and ICU mortality in diabetic patients. B:Subgroup analyses of the association between the stress hyperglycemia ratio (SHR) and 28-day mortality in diabetic patients. Forest plots show hazard ratios (HRs) and 95% confidence intervals (CIs) across different clinical subgroups. No significant interactions were observed. (DOCX) [file pone.0344961.s004.docx]

## Supplementary Figure


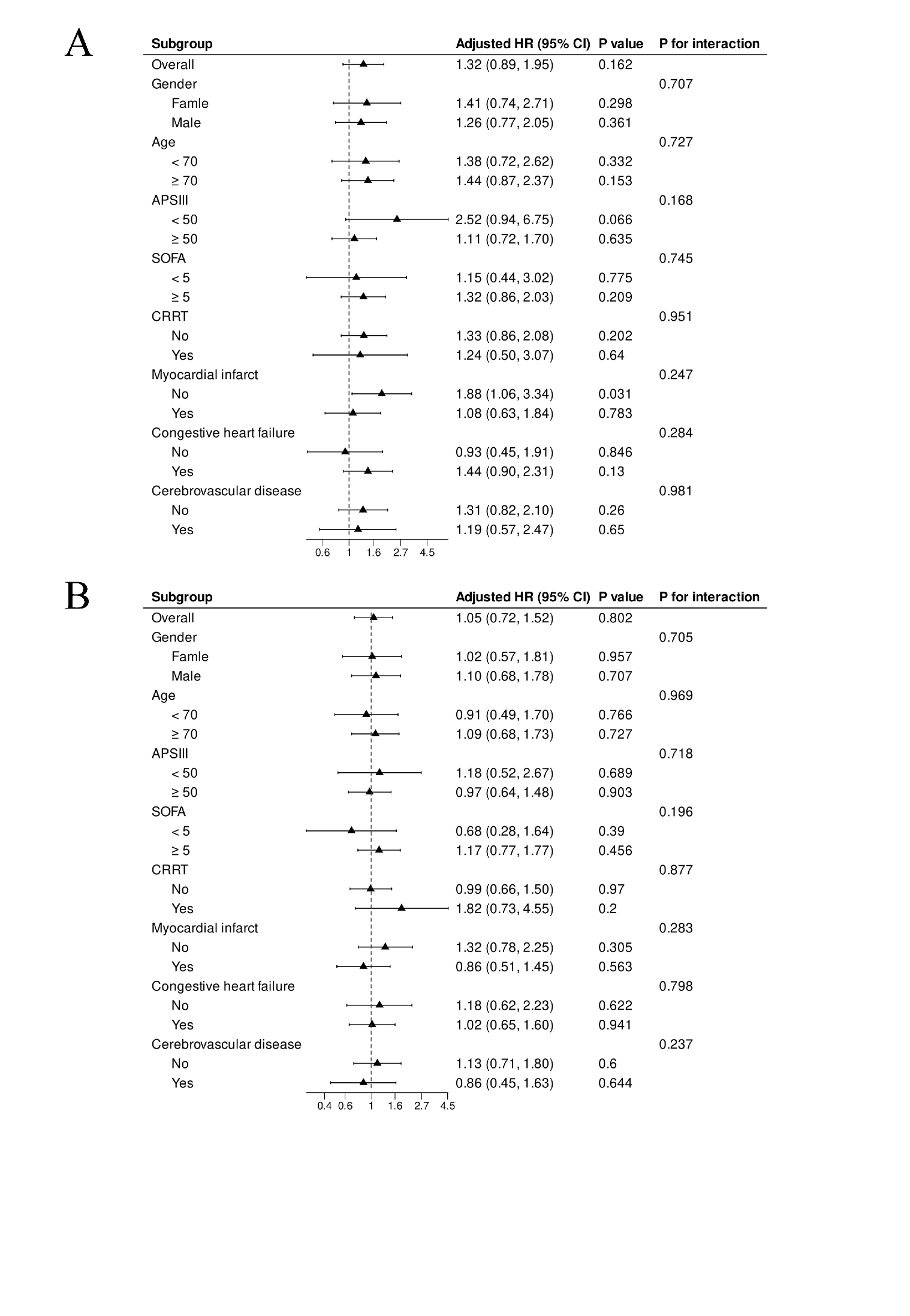


**Supplementary Figure S1:**Subgroup analyses of the association between the stress hyperglycemia ratio (SHR) and ICU and 28-day mortality in diabetic patients.

Forest plots show hazard ratios (HRs) and 95% confidence intervals (CIs) across different clinical subgroups. No significant interactions were observed.
